# *Bothrops moojeni* snake venom induces an inflammatory response in preadipocytes: Insights into a new aspect of envenomation

**DOI:** 10.1371/journal.pntd.0010658

**Published:** 2022-08-08

**Authors:** Rodrigo Maia-Marques, Danilo Santos Teixeira, Priscila Motta Janovits, Carlos DeOcesano-Pereira, Elbio Leiguez, Catarina Teixeira

**Affiliations:** 1 Laboratório de Farmacologia, Instituto Butantan, São Paulo, São Paulo, Brazil; 2 Center of Excellence in New Target Discovery (CENTD), Instituto Butantan, São Paulo, São Paulo, Brazil; Liverpool School of Tropical Medicine, UNITED KINGDOM

## Abstract

*Bothrops* envenomation is a public health problem in Brazil. Despite the advances in the knowledge of the pathogenesis of systemic and local effects induced by *Bothrops* venom, the target tissues to this venom are not completely characterised. As preadipocytes are important cells of the adipose tissue and synthesize inflammatory mediators, we investigated the ability of *B*. *moojeni* snake venom (Bmv) to stimulate an inflammatory response in 3T3-L1 preadipocytes *in vitro*, focusing on (1) the release of PGE_2_, IL-6, TNF-α, MCP-1, KC, leptin and adiponectin; (2) the mechanisms involved in PGE_2_ release and (3) differentiation of these cells. Cytotoxicity of Bmv was determined by MTT assay. The concentrations of PGE_2_, cytokines and adipokines were quantified by EIA. Participation of the COX-1 and COX-2 enzymes, NF-κB and PGE_2_ receptors (EP1-4) was assessed using a pharmacological approach, and protein expression of the COX enzymes and P-NF-κB was analysed by western blotting. Preadipocyte differentiation was quantified by Oil Red O staining. Bmv (1 μg/mL) induced release of PGE_2_, IL-6 and KC and increased expression of COX-2 in preadipocytes. Basal levels of TNF-α, MCP-1, leptin and adiponectin were not modified. Treatment of cells with SC560 (COX-1 inhibitor) and NS398 (COX-2 inhibitor) inhibited Bmv-induced PGE_2_ release. Bmv induced phosphorylation of NF-κB, and treatment of the cells with TPCK and SN50, which inhibit distinct NF-κB domains, significantly reduced Bmv-induced PGE_2_ release, as did the treatment with an antagonist of PGE_2_ receptor EP1, unlike treatment with antagonists of EP2, EP3 or EP4. Bmv also induced lipid accumulation in differentiating cells. These results demonstrate that Bmv can activate an inflammatory response in preadipocytes by inducing the release of inflammatory mediators; that PGE_2_ production is mediated by the COX-1, COX-2 and NF-κB pathways; and that engagement of EP1 potentiates PGE_2_ synthesis via a positive feedback mechanism. Our findings highlight the role of the adipose tissue as another target for Bmv and suggest that it contributes to *Bothrops* envenomation by producing inflammatory mediators.

## Author summary

Snakes of the genus *Bothrops* are responsible for most snakebite accidents in Latin America. *Bothrops moojeni*, a venomous member of this genus, is of particular medical importance in Brazil. Despite significant advances in the study of the pathogenesis of systemic and local effects induced by *Bothrops* venom, the target tissues and their responses to envenomation are poorly understood. We investigated the effects of *B*. *moojeni* venom (Bmv) on preadipocytes, focusing on the release of inflammatory mediators, lipid accumulation and mechanisms involved in PGE_2_ production. Our results show that Bmv induced an inflammatory response in preadipocytes, with activation of the NF-κB signalling pathway, release of the inflammatory mediators PGE_2_, IL-6 and KC and expression of COX-2. Furthermore, we show that engagement of the EP1 receptor potentiates PGE_2_ biosynthesis by positive feedback. Bmv also induced lipid accumulation in differentiating preadipocytes. These results indicate that adipose tissue cells may be another target for Bmv and highlight the importance of the adipose tissue as a source of inflammatory mediators during *Bothrops* envenomation, providing a new perspective for studies of this envenomation. Besides contributing to systemic effects, the mediators released by adipose tissue cells following activation by *Bothrops* venom may intensify local effects.

## Introduction

Snakebites are an important public health issue in tropical regions of the world and are considered a neglected disease by the WHO [1]. The genus *Bothrops* is responsible for most snakebites in Brazil, and the species *B*. *moojeni* is responsible for most snakebites in the Cerrado of southeastern and central Brazil, particularly in Minas Gerais and São Paulo, the two states with the highest human population in the country [2,3]. Clinically, *Bothrops* envenomation is characterised by local effects, such as oedema, inflammatory pain, haemorrhage and myonecrosis [4,5], and severe systemic effects, including coagulation disturbances, hypotension and renal failure [6,7]. The systemic effects indicate that *Bothrops* venom reaches blood circulation and triggers deleterious events in many tissues in the bitten victims. In this context the adipose tissue should be considered, since it is widely distributed throughout the body, has diverse physiological functions and impacts a wide variety of body systems [8,9].

Classically described as an energy reservoir that stores lipids, the adipose tissue is now recognized as an endocrine organ that participates in a wide variety of physiological and pathological processes [8,10]. Studies demonstrating the ability of this tissue to secrete several factors that play a role in immunological responses have shown its close association with a number of inflammatory diseases, such as rheumatoid arthritis, type II diabetes, obesity [11–14] and recently the SARS-CoV-2-induced inflammatory storm [15–19].

The adipose tissue is composed mainly of mature adipocytes and the stromal vascular fraction. The latter comprises endothelial cells, leukocytes (macrophages and lymphocytes), fibroblasts and preadipocytes [20]. Preadipocytes are fibroblast-like cells with proinflammatory features that can acquire a macrophage-like phenotype, displaying phagocytic and antimicrobial activities [21–23]. Preadipocytes and adipocytes *per se* are able to produce and release a vast array of inflammatory mediators, such as prostaglandins [24,25], cytokines, chemokines [10,26,27] and inflammatory mediators specifically secreted by the adipose tissue known as adipokines, including leptin, resistin and adiponectin [10].

Prostaglandin E_2_ (PGE_2_) is one of the prostanoids released by the adipose tissue. This lipid mediator is involved in many physiological functions, but also in the inflammatory response, as it mediates vasodilation, oedema formation and hyperalgesia [28]. Moreover, vasodilation triggered by PGE_2_ may lead to systemic hypotension in certain inflammatory conditions [29,30]. Synthesis of this mediator begins when phospholipases A_2_ act on membrane phospholipids, releasing free arachidonic acid [31], which in turn is processed by the cyclooxygenase (COX) enzyme system. This is followed by the activity of terminal synthases (PGES). PGE_2_ exerts its effects through four subtypes of G protein-coupled receptors: EP1, EP2, EP3 and EP4 [32]. In the adipose tissue, PGE_2_ is involved in maturation of preadipocytes and modulation of the release of inflammatory mediators such as leptin [33,34] via engagement of EP receptors [35–37]. In line with this, several species of *Bothrops* snake venoms have been reported to induce the release of PGE_2_ in different *in vivo* and *in vitro* experimental models [38–41]. However, to date, the effects of these venoms on the release of prostaglandins and cytokines by adipose tissue cells are still unknown.

We therefore hypothesized that the adipose tissue can be targeted by *B*. *moojeni* snake venom and can be a source of inflammatory mediators during envenomation. Based on this working hypothesis and in view of the fact that preadipocytes are cells with proinflammatory potential, we investigated the ability of *B*. *moojeni* whole venom (Bmv) to activate preadipocytes in culture, focusing on (1) the release of PGE_2_, IL-6, TNF-α, MCP-1, KC, leptin and adiponectin; (2) the mechanisms involved in PGE_2_ release; and (3) differentiation of these cells. We show that Bmv is able to stimulate preadipocytes to release PGE_2_ by activation of the COX-1 and COX-2 pathways with participation of the transcription factor NF-κB.

## Material and methods

### Venom, chemicals and reagents

*Bothrops moojeni* venom was collected, lyophilized, identified and provided by the Herpetology Laboratory of Instituto Butantan. The venom batches used were tested for endotoxin contamination using the quantitative limulus amoebocyte lysate (LAL) test [42], which revealed undetectable levels of endotoxin (<0.125 EU/mL). The venom was reconstituted in sterile PBS and immediately filtered before use. L-glutamine was purchased from USB (Cleveland, OH, USA). Dulbecco’s Modified Eagle Medium (DMEM) and Foetal Bovine Serum (FBS) were purchased from Life Technologies (São Paulo, SP, Brazil); gentamicin was purchased from Schering-Plough (Whitehouse Station, NJ, USA); insulin, 3-isobutyl-1-methylxanthine (IBMX), dexamethasone, rosiglitazone, 3-(4,5-dimethylthiazol-2-yl)-2,5-diphenyltetrazolium bromide (MTT), dimethyl sulfoxide (DMSO) and mouse anti-β-actin monoclonal antibody were purchased from Sigma-Aldrich (St. Louis, MO, USA); and polyclonal antibody against COX-1, the PGE_2_ enzyme immunoassay kit, SC-560, NS-398, SC-19220, AH6890, L-798106 and GW 627368X were purchased from Cayman Chemical Company (Ann Arbor, MI, USA). CellTrace CFSE Cell Proliferation Kit (Molecular Probes, C34554) was purchased from Life Technologies (Eugene, Oregon, USA). Polyclonal antibody against COX-2, HRP-conjugated anti-mouse secondary antibody and IL-6, KC, MCP-1, TNF-α, leptin and adiponectin EIA kits were purchased from Thermo Fisher (Waltham, Massachusetts, USA). Monoclonal antibody against phosphorylated NF-κB (P-NF-κB) and native NF-κB were purchased from Cell Signalling Technologies (Danver, Massachusetts, USA). HRP-conjugated anti-rabbit secondary antibody and nitrocellulose membrane were purchased from GE Healthcare (Buckinghamshire, UK).

### 3T3-L1 cell culture

3T3-L1 murine preadipocytes obtained from the American Type Culture Collection were cultured in Dulbecco’s Modified Eagle Medium (DMEM) supplemented with FBS, 10% (v/v) until confluence. Before stimulation with Bmv, FBS was replaced by Bovine Serum Albumin (BSA) 0.2%.

### 3T3-L1 preadipocyte differentiation

A preadipocyte differentiation assay was performed according to a previously established protocol [43]. Briefly, 1.5 x 10^4^ 3T3-L1 preadipocytes were added to each well in 24-well plates and cultured to 100% confluence (2 x 10^5^ cells/well). Two experimental groups were then defined: T0, consisting of preadipocytes with total confluence and no stimuli, and T2, consisting of preadipocytes treated with DMEM without antibiotic, supplemented with a high concentration of glucose (4500 mg/L), FBS (10%) and L-glutamine (1%), plus the differentiation cocktail (5 μg/mL insulin, 0.5 mM IBMX, 1 μM dexamethasone and 2 μM rosiglitazone) (control subgroup), or the same medium with the addition of Bmv (1 μg/mL) for 24 h (Bmv-treated subgroup). In T2, the culture medium of both subgroups was replaced by DMEM with a high glucose concentration and the differentiation cocktail without Bmv for an additional 24 h. Lipid accumulation was quantified as previously described [43].

### Cytotoxicity assay

The effects of Bmv and pharmacological compounds on cell viability of 3T3-L1 preadipocytes were evaluated using the MTT assay [44,45]. Briefly, preadipocytes were incubated at 37°C in a humidified atmosphere (5% CO_2_) with DMEM supplemented with FBS (10%), L-glutamine (1%) and gentamicin sulphate and incubated with different concentrations of Bmv (0.5, 1 or 5 μg/mL), or the pharmacological compounds or with either DMEM (with 0.2% BSA) as a negative control or DMEM with Triton 10% as a positive control for 1, 3, 6 and 24 h. MTT (5 mg/mL) was dissolved in PBS and filtered for sterilization and removal of insoluble residues. Next, the cells were incubated with medium containing MTT (10%) for 2 hours. DMSO (250 μL) was then added to each well and mixed thoroughly for 30 min at room temperature. Absorbances were recorded at 540 nm in a spectrophotometer. The results were expressed as percentages of viable cells, and negative control cells were considered 100% viable.

### Cell proliferation assay

The effects of Bmv on proliferation of 3T3-L1 preadipocytes were evaluated using a commercially available Cell Trace CFSE Cell Proliferation Kit (Life Tech). Cells were labeled according to the manufacturer’s instructions. Labelled cells were seeded in black advanced TC 96-well microplates at the density of 1x10^3^ cells/well, kept in culture in 10% FBS DMEM for 48 h and then incubated with Bmv (0.5 or 1 μg/mL) or DMEM for 24 h. Cells not labelled with CFSE were used as a background control. Afterwards, cells were fixed with cold 4% paraformaldehyde for 1 h. The High-Content Screening (HCS) assay was used to assess the fluorescence of single cells from a cell population 24 h after treatment [46]. Then, the cells nuclei were stained with Hoechst-33342 (Thermo Fisher Scientific, H3570) for 30 min at room temperature. Cell quantification based on images was performed using MetaXpress software (Molecular Devices, USA). An internal mask (cytoplasm) was defined by dilating the nuclear mask out to the edge of the Hoechst-33342. The fluorescence intensity parameters of the CFSE were measured inside the cytoplasm area (FITC channel). The quantitative data obtained represent median fluorescence intensity of the CFSE marker (median 16 sites per well) relative to the negative control (cells not labelled with CFSE or cells with CFSE without the Bmv).

### Quantification of PGE_2_, cytokines and adipokines

Quantification of PGE_2_, cytokines (IL-6, TNF-α, KC and MCP-1) and adipokines (leptin and adiponectin) was performed in the supernatants collected from cell cultures by enzyme immunoassay (EIA) using a commercially available kit (Cayman Chemicals, ThermoFisher). The tests were performed according to the supplier’s specifications. Concentrations were estimated from the standard curve and represented in pg/mL.

### Pharmacological interventions

To evaluate the participation of COX-1, COX-2, NF-κB and each PGE_2_ receptor subtype in the Bmv-induced effects, pharmacological interventions were performed with selective inhibitors or antagonists in concentrations described in the literature [47–52]: 1 μM SC-560 (COX-1 inhibitor, 1 h before Bmv); 1 μM NS-398 (COX-2 inhibitor, 1 h before Bmv); 10 μM SC-19220 (EP1 receptor antagonist, 1 h before Bmv); 10 μM AH 6809 (EP2 receptor antagonist, 1 h before Bmv); 1 μM L-798,106 (EP3 receptor antagonist, 1 h before Bmv); 10 μM GW 627368X (EP4 receptor antagonist, 1 h before Bmv); 30 μM TPCK (NF-κB inhibitor, 24 h before Bmv); 50 μg / mL SN50 (NF-κB inhibitor, 2 h before Bmv). Some of the used compounds were prepared in DMSO at concentration lower than 1%. Cells treated with the inhibitors were analysed for viability by the MTT colorimetric assay. No significant changes in cell viability were registered with any of the above agents or vehicles at the concentrations used (S1 Fig).

### Western blotting

The protein content of COX-1, COX-2 and P-NF-κB was determined in cell lysates by western blotting. For this purpose, the cells incubated or not with Bmv were lysed by adding 100 μL/well of Laemmli buffer (0.5 M Tris-HCl, 20% SDS, 1% glycerol, 1 M β-mercaptoethanol, 0.1% bromophenol blue) and boiled for 10 min. Samples were resolved by SDS-PAGE (12% bis-acrylamide gels) electrophoresis. The proteins were transferred to a nitrocellulose membrane with a Mini Trans-Blot (Bio-Rad Laboratories, Richmond, CA, USA). The membranes were blocked for 1 h with 5% albumin in Tris-buffered saline Tween 20 (20 mM Tris, 100 mM NaCl and 0.5% Tween 20, pH 7.2) and incubated overnight at 4°C with COX-1, COX-2, P-NFκB or NF-κB primary antibodies (1:1000 dilution) and for 1 h at room temperature with the β-actin primary antibody (1:3000 dilution). Next, the membranes were washed and incubated with the appropriate secondary antibody conjugated to horseradish peroxidase. Immunoreactive bands were detected using an entry-level peroxidase substrate for enhanced chemiluminescence (Pierce ECL Western Blotting Substrate) according to the manufacturer’s instructions (Thermo Fisher Scientific, Waltham, MA, USA). Band images were captured with an ImageQuant LAS 4000 mini biomolecular imager (GE Healthcare) and analysed with ImageQuant TL software (GE Healthcare).

### Statistical analysis

The results were expressed as mean + standard error of the mean (S.E.M.). Two-way analysis of variance (two-way ANOVA) was used, followed by multiple comparisons with the Bonferroni post-test. The normality and homoscedasticity of all samples were checked previously. The data were analysed with GraphPad Prism 8.0.1 (GraphPad, San Diego, CA, USA). A significance level of *p* < 0.05 was adopted.

## Results

### Bmv induces the release of PGE_2_ by preadipocytes in culture

**Fig 1:**
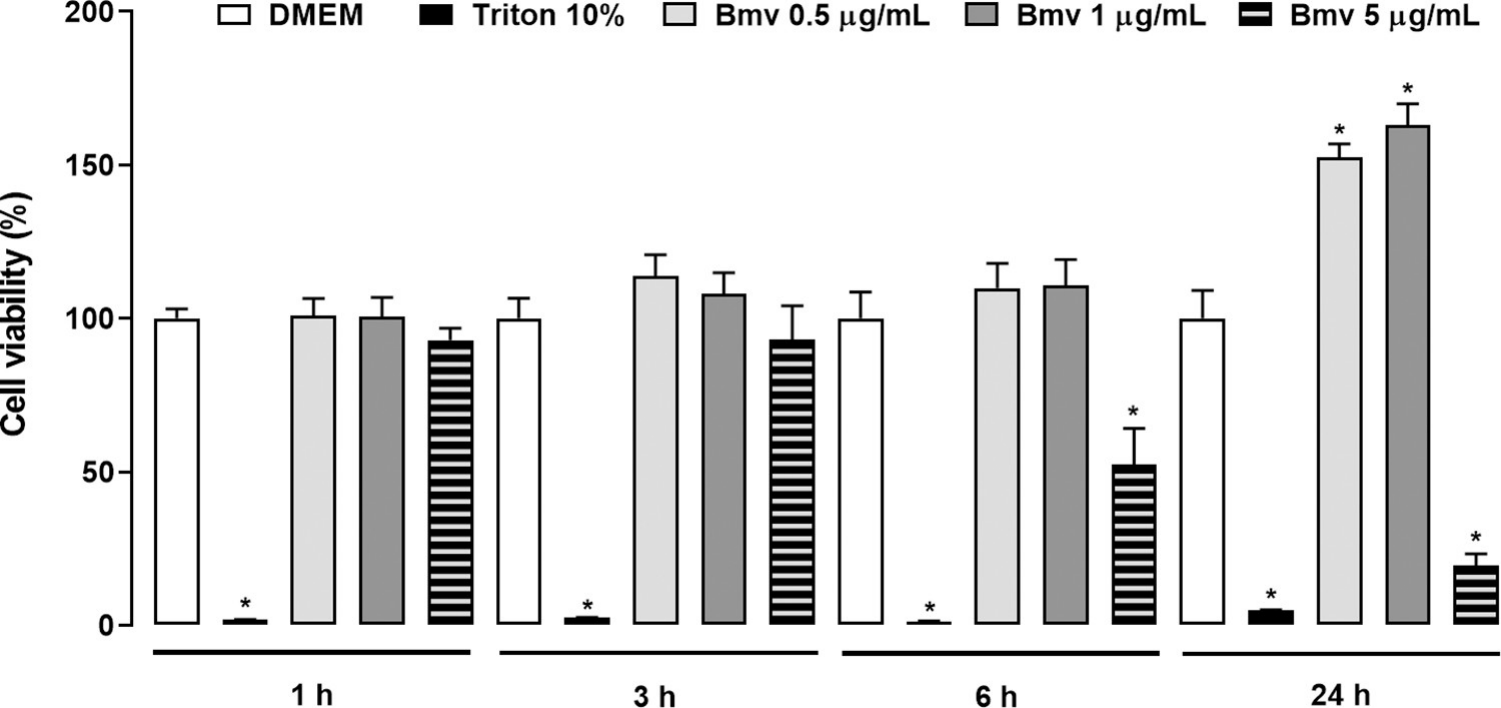
Time course of Bmv-induced effect on viability of preadipocyte cell culture. 3T3-L1 preadipocytes (2 x 10^5^ cells/well) were incubated with Bmv (0.5–5 μg/mL) or DMEM (control) for 1, 3, 6 and 24 h. Metabolic activity was assessed by the MTT assay. Results are expressed as mean + S.E.M. of 3 independent assays (n = 4). *p < 0.05 *vs* control (ANOVA and Bonferroni post test).

**Fig 2:**
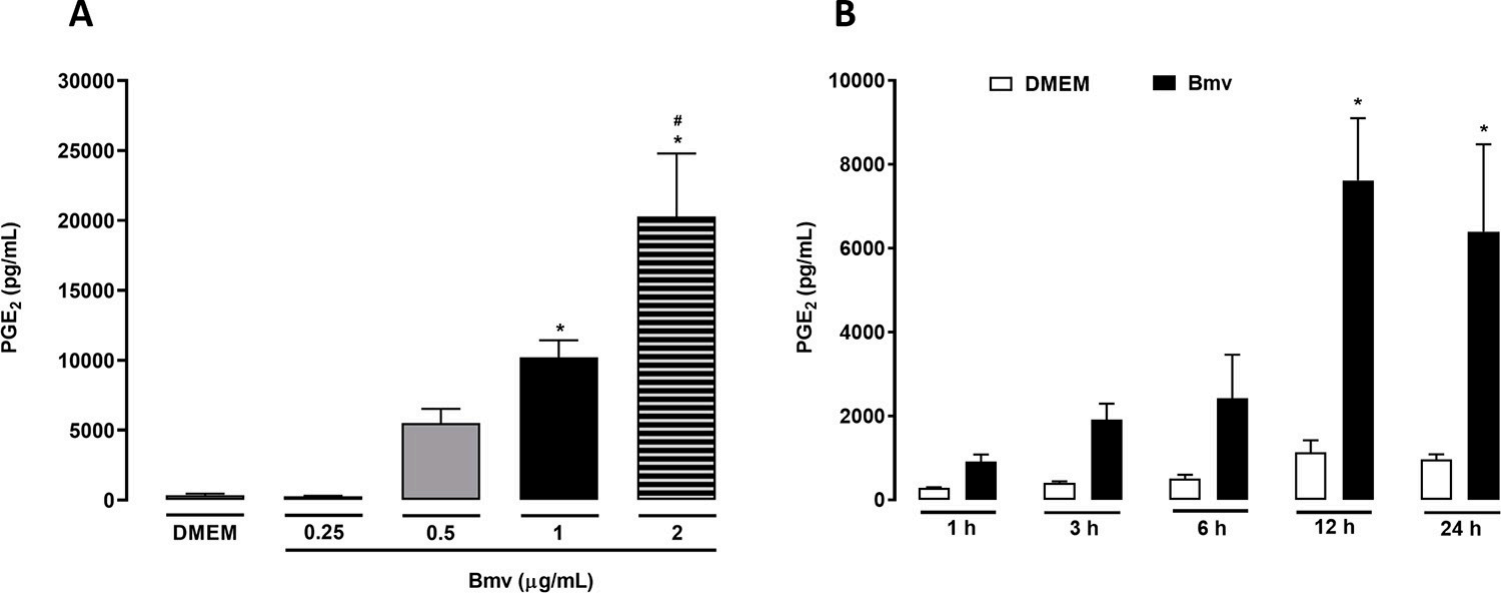
Bmv stimulates the release of PGE_2_ by preadipocytes. (**A**) Release of PGE_2_ induced by selected concentrations of Bmv. 3T3-L1 preadipocytes (2 x 10^5^ cells/well) were incubated with Bmv at distinct concentrations (indicated above) or DMEM (control) for 24 h. Results are expressed as mean + S.E.M. (n = 4). *p < 0.05 *vs* respective control (DMEM); ^#^*p* < 0.05 vs Bmv 1 μg/mL group (unpaired t test). (**B**) Time course of Bmv-induced synthesis of PGE_2_. 3T3-L1 preadipocytes (2 x 10^5^ cells/well) were incubated with Bmv (1 μg/mL) or DMEM (control) for the indicated time intervals. Results are expressed as mean + S.E.M. of 3 independent assays (n = 4). *p < 0.05 *vs* negative control (DMEM) (ANOVA and Bonferroni post test). Concentration of PGE_2_ in culture supernatants was evaluated by EIA in both experiments.

PGE_2_ is an important mediator of inflammatory and hyperalgesic processes [53], and previous studies have demonstrated that the venom of some species of *Bothrops* snakes induces the release of this mediator at the injection site [40,54,55]. Although PGE_2_ is known to be one of the most abundant lipid mediator produced in the adipose tissue [56], to date it is not known whether the venom of *B*. *moojeni* can activate this tissue to release prostaglandins. We therefore decided to investigate the extent to which Bmv can promote the release of PGE_2_ by preadipocytes in culture. We carried out preliminary assays, which demonstrated that at concentrations between 0.5 and 1 μg/mL, Bmv does not affect cell viability from 1 to 24 h of incubation (Fig 1). Based on these data, the maximal non-cytotoxic concentration (1 μg/mL) was used. At this concentration, Bmv did not induce cell proliferation (S2 Fig). Bmv (1 μg/mL) was added to the culture medium for 1, 3, 6, 12 and 24 h, and PGE_2_ release was quantified by EIA. As shown in Fig 2A, incubation of preadipocytes with Bmv at concentrations of 1 and 2 μg/mL, but not 0.25 and 0.5 μg/mL, resulted in PGE_2_ levels significantly higher than those observed in control cells incubated with culture medium alone after 24 h. Fig 2B shows that stimulation of preadipocytes with Bmv at 1 μg/mL induced significant release of PGE_2_ from 12 to 24 h, but not at the earlier time intervals evaluated compared with control-group cells. This result shows that Bmv can stimulate preadipocytes to synthesize PGE_2_ in a concentration- and time-dependent manner.

### COX-1 and COX-2 participate in Bmv-induced PGE_2_ release by preadipocytes

**Fig 3:**
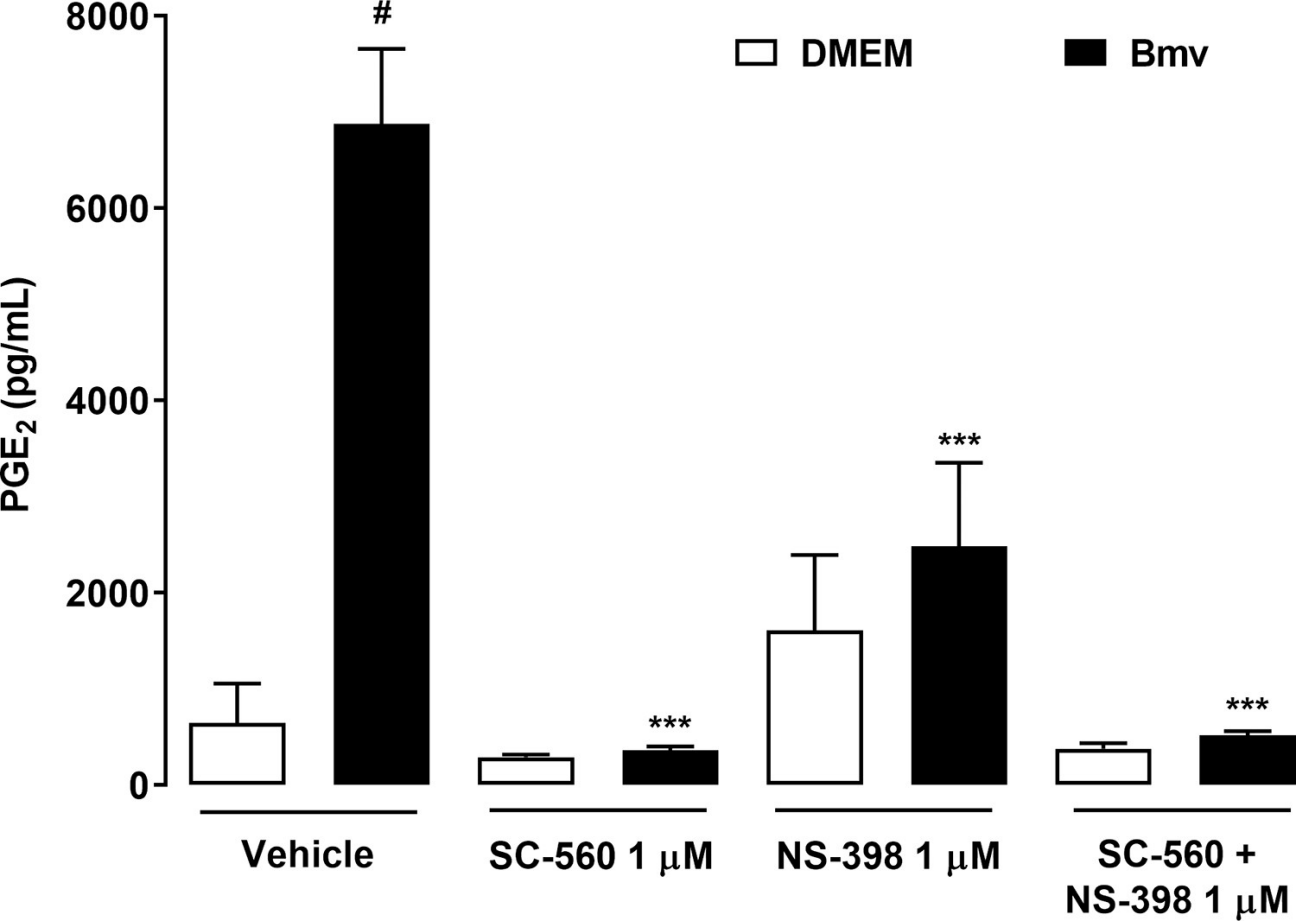
COX-1 and COX-2 participate in Bmv-induced PGE_2_ release by preadipocytes. 3T3-L1 preadipocytes were pretreated with COX-1 and COX-2 selective inhibitors SC-560 and NS-398, respectively, or vehicle (DMSO < 1%) for 1 h and then stimulated with Bmv (1 μg/mL) or DMEM (control) for 24 h. Concentration of PGE_2_ present in supernatants was quantified by EIA. Results are expressed as mean + SEM (n = 4). ^#^*p* < 0.05 vs. negative control (vehicle + DMEM); ***p < 0.001 vs. positive control (vehicle + Bmv) (ANOVA and Bonferroni post test).

**Fig 4:**
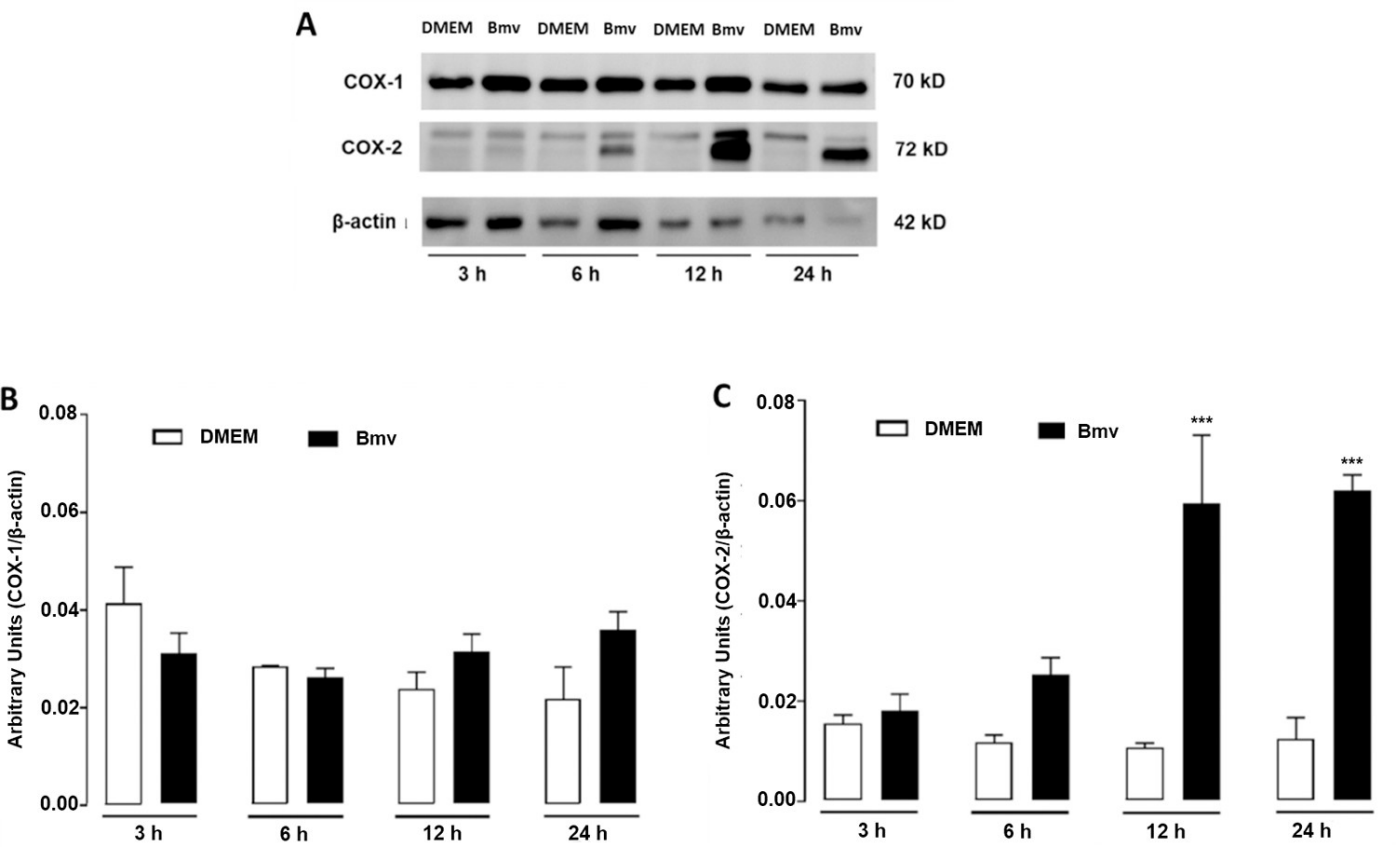
Bmv upregulates COX-2 protein expression in preadipocytes. 3T3-L1 preadipocytes were stimulated with Bmv (1 μg/mL) or DMEM (control) for 3, 6, 12 or 24 h. COX-1 and COX-2 protein expression was evaluated by western blotting. (A) Representative immunoreactive bands for COX-1, COX-2 and β-actin (loading control). Densitometric analysis of immunoreactive bands of (B) COX-1 and (C) COX-2. Results are expressed as mean + SEM (n = 4). ***p < 0.001 vs. control (ANOVA and Bonferroni post test).

COX enzymes are crucial for the synthesis of PGE_2_ from arachidonic acid in inflammatory processes [57]. Although both COX-1 and COX-2 are found constitutively in different tissues, COX-2 is inducible in inflammatory conditions and in cells of the adipose tissue [25,58,59]. To investigate the mechanisms underlying PGE_2_ production induced by Bmv, we evaluated the participation of COX-1 and COX-2 isoforms in Bmv-induced PGE_2_ release using pharmacological approaches. Preadipocytes were treated with either COX-1 or COX-2 selective inhibitors SC-560 and NS-398, respectively, or with vehicle (DMSO < 1%) for 1 h before stimulation with Bmv (1 μg/mL) or DMEM (control) for 24 h. PGE_2_ release was then evaluated in the culture supernatants. As shown in Fig 3, preadipocytes preincubated with vehicle followed by stimulation with Bmv showed significant release of PGE_2_ compared with non-stimulated preadipocytes (basal control). Bmv-induced PGE_2_ release was abolished in preadipocytes pretreated with SC-560 but not in preadipocytes treated with vehicle followed by stimulation with venom (positive control). Pretreatment of cells with NS-398 markedly decreased Bmv-induced PGE_2_ release in comparison with the positive control. In addition, pretreatment of preadipocytes with both SC-560 and NS-398 abolished Bmv-induced PGE_2_ release, which was still observed in cells without this pretreatment (positive control). This is a strong evidence that COX-1 and COX-2 play a role in Bmv-induced production of PGE_2_ in preadipocytes. Based on these findings, we further investigated whether Bmv can induce COX-2 protein expression in preadipocytes. As shown in Fig 4A and 4B, incubation of preadipocytes with Bmv did not change protein expression of the constitutive isoform COX-1 at the time points assessed, but increased COX-2 protein expression was observed at the 12 h and 24 h time points (Fig 4A and 4C). Altogether, these results suggest that Bmv-induced PGE_2_ synthesis depends on both COX-1 and COX-2 signalling pathways. Furthermore, these data point to the ability of this venom to upregulate COX-2 protein expression, a mechanism leading to PGE_2_ release after longer incubation times.

### NF-κB is activated by Bmv and regulates Bmv-induced PGE_2_ release in preadipocytes

**Fig 5:**
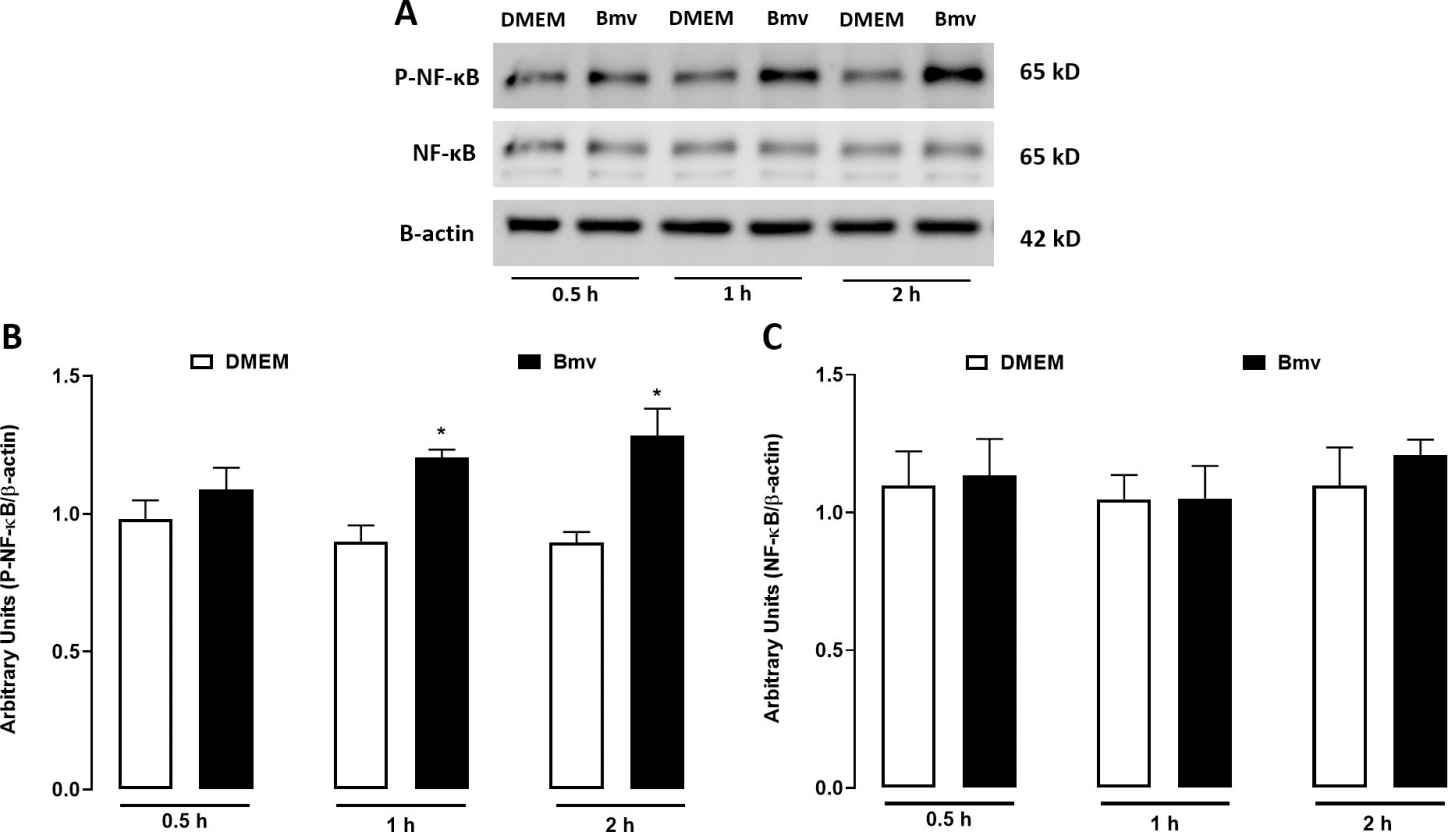
Bmv activates NF-κB in preadipocytes. 3T3-L1 preadipocytes were stimulated with Bmv (1 μg/mL) or DMEM (control) for 0.5, 1, or 2 h. P-NF-κB and NF-κB protein expression was evaluated by western blotting. (A) Representative immunoreactive bands for P-NF-κB, NF-κB and β-actin (loading control). Densitometric analysis of immunoreactive bands of (B) P-NF-κB and (C) native NF-κB. Results are expressed as mean + SEM (n = 3). *p < 0.05 vs. control (ANOVA and Bonferroni post test).

**Fig 6:**
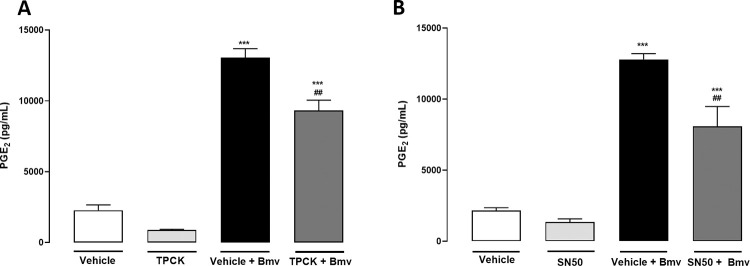
NF-κB participates in Bmv-induced PGE_2_ release by preadipocytes. 3T3-L1 preadipocytes were pretreated with NF-κB inhibitors (A) TPCK or vehicle, for 24 h or (B) SN50 or vehicle (DMSO < 1%) for 1 h, and then stimulated with Bmv (1 μg/mL) or DMEM (control) for 24 h. Concentration of PGE_2_ present in supernatants was quantified by EIA. Results are expressed as mean + SEM (n = 4). ***p < 0.001 vs. negative control (vehicle + DMEM); ^##^*p* < 0.01 vs. positive control (vehicle + Bmv) (ANOVA and Bonferroni post test).

Several inflammatory stimuli, such as TLR ligands and cytokines, can activate NF-κB [60], a major transcription factor that regulates a number of inflammatory genes, including those coding for enzymes that play a critical role in the PGE_2_ biosynthesis pathway [61–63]. To determine possible mechanisms involved in the inflammatory effects of Bmv in preadipocytes, we investigated the ability of this venom to activate NF-κB. The cells were stimulated with Bmv for 0.5, 1 and 2 h, and the phosphorylated-NF-κB (P-NF-κB) p65 subunit and native NF-κB were quantified by western blotting. Fig 5A shows representative immunoreactive bands of P-NF-κB, native NF-κB and β-actin. Fig 5B shows the densitometric analysis of the band intensities normalized with those of β-actin. P-NF-κB protein levels did not change after 0.5 h of Bmv stimulation in comparison with the negative control group. However, after the 1 h and 2 h time points, preadipocytes stimulated with Bmv showed an increase in P-NF-κB protein expression in comparison with the negative control group. Native NF-κB did not show marked changes in its expression levels upon Bmv stimulus compared with the actin loading control (Fig 5C). To further elucidate the mechanisms of Bmv-induced PGE_2_ release by preadipocytes, we investigated the participation of NF-κB in this event using a pharmacological approach. Preadipocytes were treated with TPCK, an inhibitor of both IKKβ and the p65 subunit of the NF-κB activation pathway [50], or vehicle (DMSO < 1%) for 24 h prior to the Bmv stimulus, or SN50, a NF-κB nuclear translocation inhibitor [64], or vehicle (DMSO < 1%) for 1 h before the Bmv stimulus. In both treatment protocols, PGE_2_ release was measured 24 h after the stimulus. Fig 6 shows that preadipocytes preincubated with vehicle followed by stimulation with Bmv showed significant release of PGE_2_ compared with non-stimulated preadipocytes (basal control). Treatment of the cells with TPCK, a two-step inhibitor which acts on the p65 subunit of NF-κB, before stimulation with Bmv, significantly reduced the release of PGE_2_ in comparison with cells pretreated with vehicle only and stimulated with Bmv. Similarly, pretreatment of cells with SN50, a competitive inhibitor of the p50 NF-κB subunit involved in nuclear translocation, significantly reduced Bmv-induced PGE_2_ levels in comparison with control cells pretreated with vehicle only and stimulated with Bmv. Altogether, these results indicate (1) that Bmv activates the NF-κB pathway in preadipocytes and (2) that NF-κB is involved in the pathway triggered by Bmv that leads to PGE_2_ production in preadipocytes.

### EP1 receptor participates in Bmv-induced PGE_2_ release by preadipocytes

**Fig 7:**
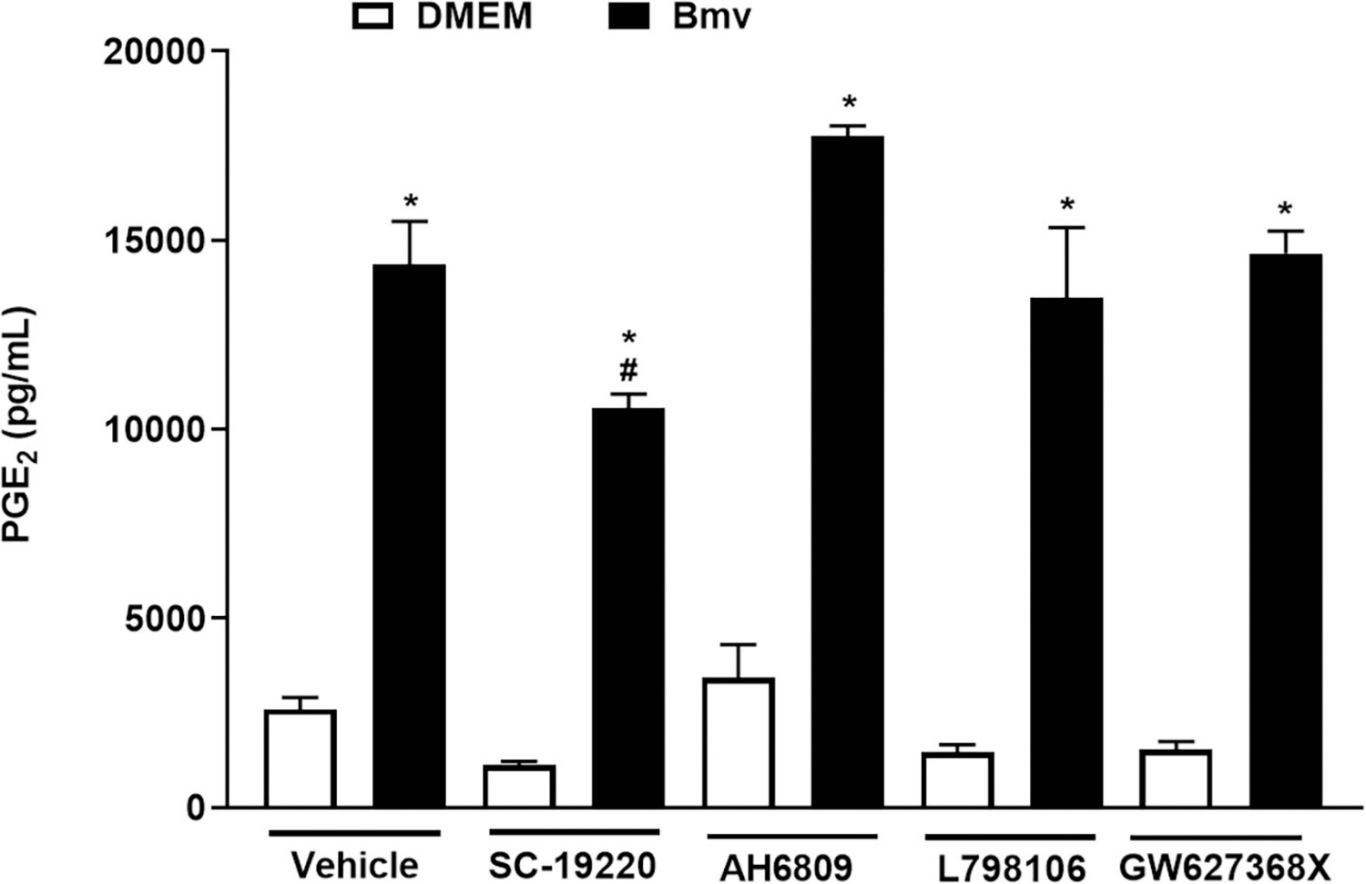
EP1, but not EP2-4 subtype receptors, participates in Bmv-induced PGE_2_ release by preadipocytes. Preadipocytes were incubated with the EP receptors antagonists SC-19220 (EP1 receptor antagonist,10 μM), AH6809 (EP2 receptor antagonist,10 μM), L-798106 (EP3 receptor antagonist,1 μM) or AH23848 (EP4 receptor antagonist,10 μM) or vehicle (DMSO < 1%) for 1 h followed by stimulation with Bmv (1 μg/mL) for 24 h. Concentration of PGE_2_ present in supernatants was quantified by EIA. Results are expressed as mean + S.E.M. (n = 4). *p < 0.05 vs. negative control (vehicle + DMEM); ^#^*p* < 0.05 vs. positive control (vehicle + Bmv) (ANOVA and Bonferroni post test).

The effects of PGE_2_ are exerted by activation of four G-protein coupled receptor subtypes known as EP1-4 in a paracrine or endocrine way [65]. Besides mediating the physiological and pathophysiological effects of PGE_2_, these receptors regulate PGE_2_ biosynthesis depending on the cell type and physiological environment [65]. For this reason, we investigated the involvement of the EP receptor subtypes in the production of Bmv-induced PGE_2_ in preadipocytes. Fig 7 shows that in preadipocytes treated with vehicle and stimulated with Bmv, there was a marked release of PGE_2_ in comparison with cells from the basal group (stimulated only with vehicle). Pretreatment of cells with EP1 receptor antagonist SC-19220 significantly reduced Bmv-induced PGE_2_ release in comparison with the vehicle and Bmv group (positive control). In contrast, pretreatment with EP2, EP3 or EP4 receptor antagonists did not alter Bmv-induced PGE_2_ release by preadipocytes. These findings point to the involvement of EP1, but not EP2, EP3 or EP4 subtype receptors in Bmv-stimulated PGE_2_ production.

### Bmv induces lipid accumulation in differentiating preadipocytes

**Fig 8:**
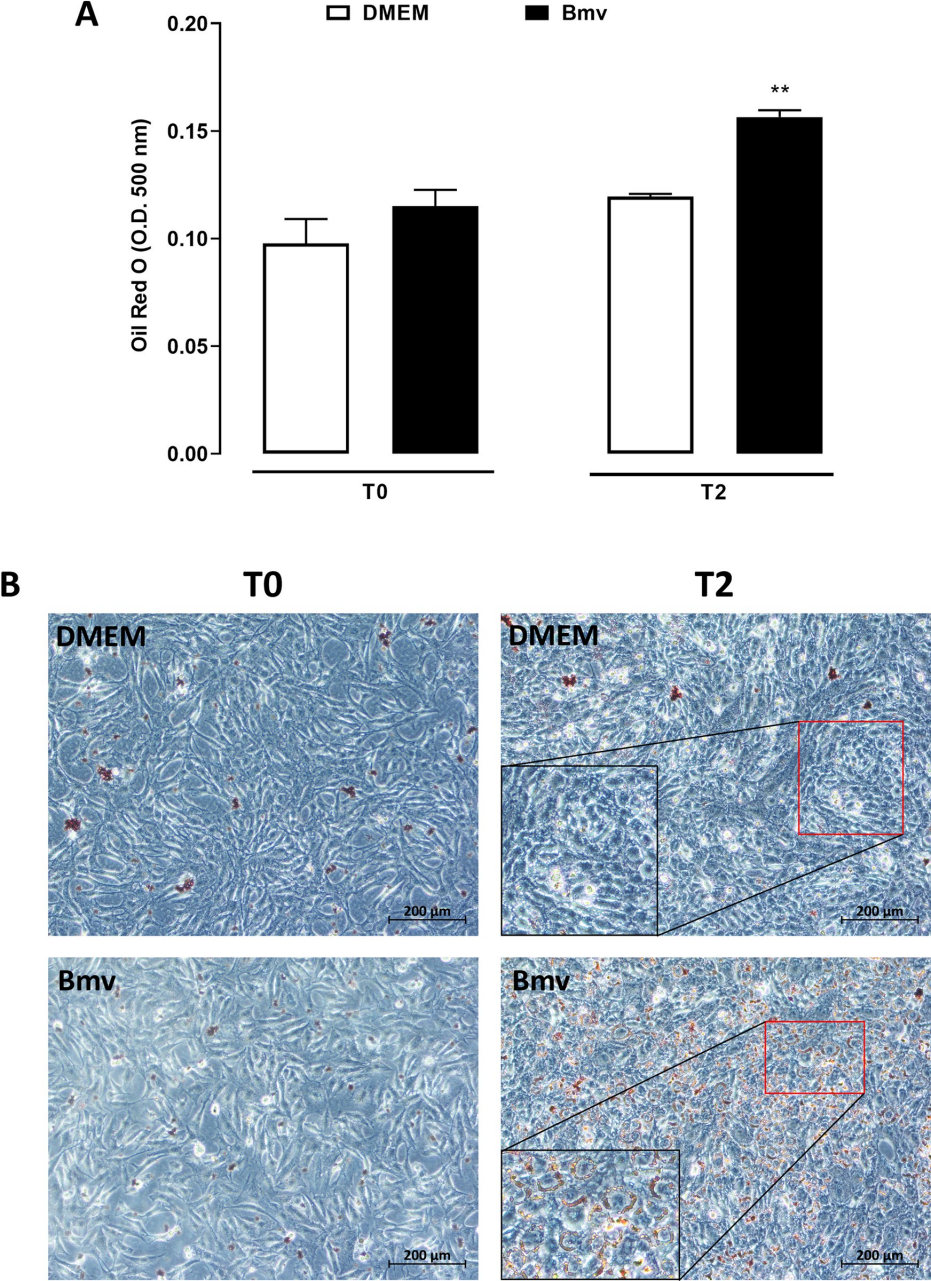
Effect of Bmv on lipid accumulation in preadipocytes. (A) Quantification of intracellular lipid content of 3T3-L1 preadipocytes subjected to differentiation medium with or without stimulation by Bmv (1 μg /mL). 24 h after stimulation, the culture medium of all groups was changed to a fresh differentiation medium, without Bmv, and maintained for 24 h. Cells were then fixed and stained with Oil Red O as described in Material and Methods. Results are expressed as mean + S.E.M. (n = 4). **p < 0.01 vs. DMEM (ANOVA and Bonferroni post test); (B) Photomicrographs of cell cultures at different experimental times and conditions. Inset in Bmv/T0 shows a high-magnification view of preadipocytes at 100% confluence without lipid accumulation. Inset in Bmv/T2 shows a high-magnification view of red-stained lipids in differentiating preadipocytes. Scale bar: 200 μm.

Adipogenesis is the differentiation of preadipocytes into mature, terminally differentiated adipocytes. The latter generally have a distinctive cellular morphology and structure, as the cells convert from fibroblastic to spherical shape and most of the cytoplasmic space is occupied by lipid droplets. This intracytoplasmic lipid accumulation is directly proportional to the extent of differentiation [66]. This relationship has been used as a qualitative marker of adipose conversion and can be determined by Oil red O staining [67]. As PGE_2_ is known to exert proliferative and antilipolytic effects on adipocytes [68], we investigated the effects of Bmv on lipid accumulation by assessing Oil Red O staining in differentiating preadipocytes. Preadipocyte cultures at 100% confluence (T0) were incubated with differentiation medium and stimulated with Bmv or DMEM (negative control) for 24 h (T1). Then, the medium of control (DMEM) and Bmv groups was replaced with a differentiation medium for an additional 24 hours (T2). Quantification of intracellular lipid content is shown in Fig 8A. In the time point T0, lipid accumulation in cells stimulated with Bmv did not differ from that observed in the control group, whereas in the time point T2, lipid accumulation was significantly increased 24 h after stimulation of cells with Bmv in comparison with non-stimulated cells. Fig 8B shows cells in the time points T0 and T2. Preadipocytes in T0 are seen at 100% confluence and without intracellular lipid deposits in both non-stimulated and Bmv-stimulated conditions. In T2, while the control preadipocytes growing in the absence of Bmv show few intracellular lipid deposits, preadipocytes stimulated with Bmv show a typical spherical shape and a visible increase in lipid content compared with the T2 control group. These findings are in line with the increased Bmv-induced release of PGE_2_ in preadipocytes. As lipid accumulation is a marker of preadipocyte differentiation into mature adipocytes, these data suggest that Bmv can stimulate preadipocyte differentiation.

### Bmv stimulates IL-6 and KC/IL-8 release by preadipocytes in culture

**Fig 9:**
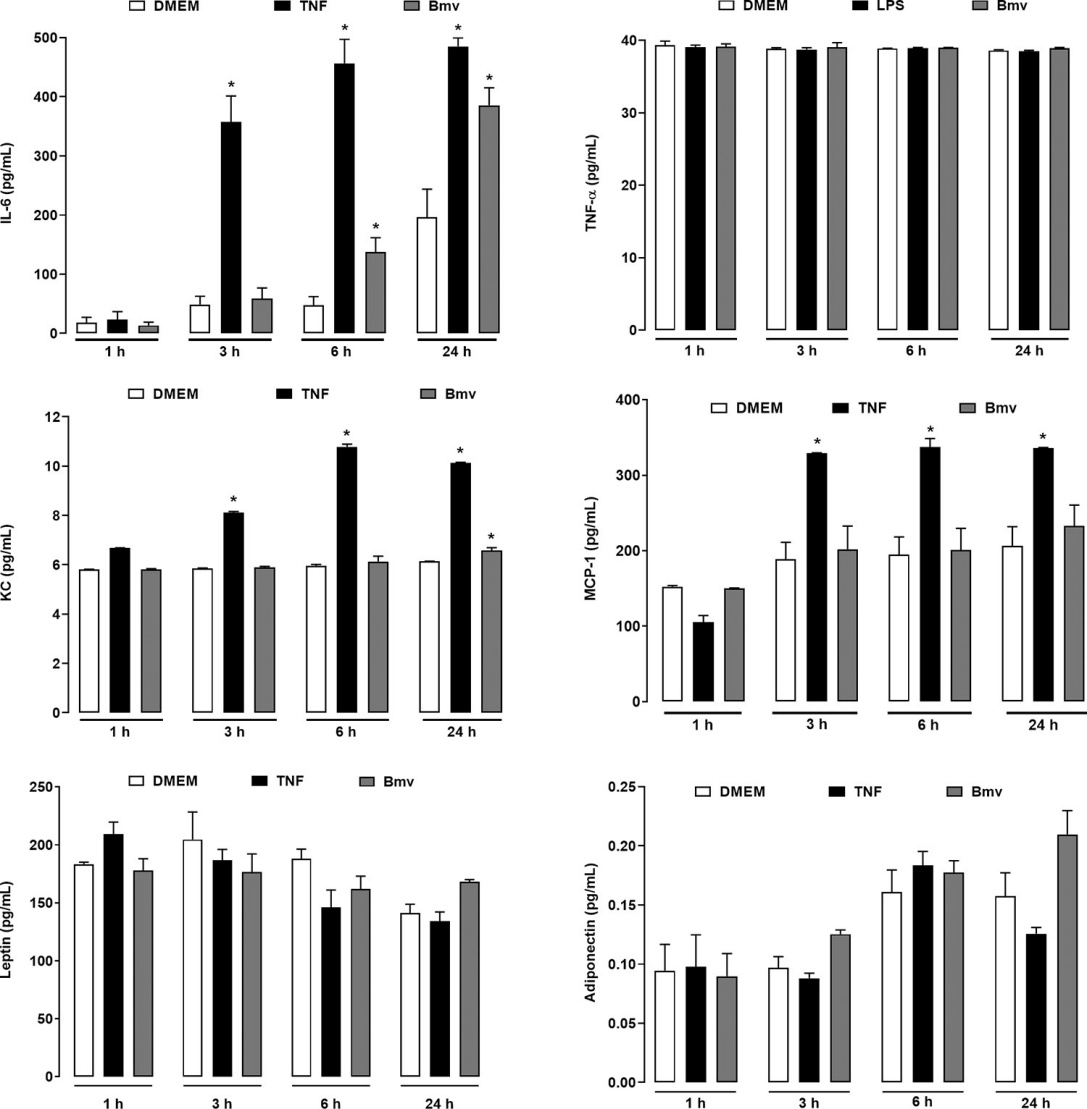
Bmv stimulates the release of IL-6 and KC/IL-8 but not TNF-α, MCP-1, leptin nor adiponectin by preadipocytes. 3T3-L1 preadipocytes (2 x 10^5^ cells/well) were incubated with Bmv (1 μg/mL), or TNF-α, 20 ng/mL (positive control) or LPS, 1 μg/mL (positive control), or DMEM (control) for the above indicated time intervals. Concentration of cytokines and adipokines in culture supernatants was evaluated by EIA. Results are expressed as mean + S.E.M. of 3 independent assays (n = 4). *p < 0.05 *vs* control (ANOVA and Bonferroni post test).

The adipose tissue was shown to produce cytokines, chemokines and adipose tissue specific mediators known as adipokines, including leptin and adiponectin [10,69]. To investigate additional effects of Bmv on adipose tissue, we evaluated Bmv-induced release of cytokines, chemokines, leptin and adiponectin by preadipocytes in culture. Bmv (1 μg/mL) was added to the culture medium for 1, 3, 6 and 24 h, and IL-6, KC/IL-8, TNF-α, MCP-1, leptin and adiponectin release was quantified by EIA. TNF-α (20 ng/mL) or LPS (1 μg/mL) were used as positive controls. As shown in Fig 9, Bmv induced significant release of IL-6 and KC/IL-8 at 6 h (IL-6) and 24 h (both cytokines) after stimulation compared with the negative control group cells incubated with culture medium alone, but not after shorter stimulation periods. In contrast, the levels of TNF-α, MCP-1, leptin and adiponectin released were the same as those released by the control cells. In the time course evaluated (24 h) LPS did not stimulate the release of TNF-α by preadipocytes.

## Discussion

The adipose tissue is able to secrete an array of substances that regulate homeostasis and immune responses and is known to contribute to the development of various inflammatory diseases. We have demonstrated in this study that Bmv can stimulate preadipocytes and induce the release of important inflammatory mediators such as PGE_2_, IL-6 and KC by these cells. To the best of our knowledge, this is the first demonstration that a snake venom can activate adipose tissue cells.

Our findings showing that Bmv induced the release of PGE_2_ by preadipocytes provide evidence of a new source of this mediator. As the adipose tissue is an endocrine organ and can contribute to inflammatory processes in distant tissues and organs, the release of PGE_2_ reported here may have an impact on the systemic effects of *Bothrops* venoms. Therefore, the role of this mediator in Bmv-induced systemic alterations deserves further investigation. PGE_2_ is known to be produced when arachidonic acid is metabolized by the enzymes COX-1 and COX-2 [68]. The former is found in most tissues [59] and is responsible for generating prostaglandins in various physiological and pathological conditions [58,70]. COX-2, in turn, can be constitutively expressed in some tissues and is also inducible under inflammatory conditions [25,40,70]. Our results showing that inhibition of COX-2 or COX-1 suppressed the Bmv-induced release of PGE_2_ indicate that both isoforms are involved in the Bmv-induced PGE_2_ biosynthetic cascade. Our data also revealed an additional mechanism implicated in the Bmv effects, namely the upregulation of COX-2 protein expression in preadipocytes. The findings reported here are in line with previous reports showing that *Bothrops* spp. venoms and toxins isolated from these venoms upregulate expression of COX-2, the inducible isoform of the COX enzymes, in immune cells [25,40,55].

COX-2 expression is regulated at both transcriptional and posttranscriptional levels. The promoter region of the COX-2 gene contains several binding sites for transcription factors such as NF-κB, CREB, C/EBP and AP-1 [71,72]. Of these, NF-κB is the main transcription factor coordinating COX-2 gene expression during inflammatory processes [73,74]. In view of this, we investigated whether Bmv activates NF-κB in preadipocytes and found that this venom increased protein expression of the P-NF-κB p65 subunit, an indicator of activation of this factor. In addition, to better understand the effects of Bmv on the COX pathway, we investigated the role of the transcription factor NF-κB in Bmv-induced PGE_2_ synthesis. Our results demonstrating that pharmacological inhibition of NF-κB with TPCK or SN50 reduced venom-induced PGE_2_ release in preadipocytes indicate that NF-κB plays a role in Bmv-induced production of PGE_2_ in these cells. As TPCK inhibits the p65 subunit and SN50 is a competitive antagonist of NF-κB that acts on the p50 subunit, it is reasonable to suggest that distinct domains of NF-κB, notably the p65 and p50 subunits, are involved in this effect of the venom. The effects observed with TPCK are in line with the results showing activation of the NF-κB p65 subunit by Bmv, highlighting the importance of this subunit for the inflammatory response triggered by this venom in preadipocytes. However, participation of other transcription factors, such as CREB, C/EBP and AP-1, in Bmv-induced effects cannot be ruled out. The upstream pathways involved in the activation of NF-κB by Bmv were not investigated here and deserve further study.

The effects of PGE_2_ are mediated by four subtypes of EP receptors (EP1-EP4) [65], and expression of these four receptors in preadipocytes has been previously reported [25]. Our data showing that the compound SC-1922, an antagonist of EP1 but not of EP2, EP3 or EP4, decreased venom-induced release of PGE_2_ indicate that activation of EP1 contributes to the increased PGE_2_ levels induced by Bmv in preadipocytes. Therefore, it is plausible to suggest that PGE_2_ engages the EP1 receptor via autocrine action and triggers a biosynthetic pathway that regulates its own production through a positive feedback loop. These findings are in line with previous reports of an EP4-dependent positive feedback loop regulating the production of PGE_2_ induced by a PLA_2_ and a metalloproteinase isolated from *B*. *asper* snake venom in different cell types [25,75].

In addition to its potent inflammatory activity, PGE_2_ exerts antilipolytic effects on adipocytes, leading to adipose tissue hypertrophy and differentiation of preadipocytes into mature adipocytes [76,77]. Hence, our findings showing that Bmv increased lipid accumulation in preadipocytes strongly suggest that this venom can induce differentiation of these cells and that PGE_2_ is involved in this effect. The molecular factors involved in preadipocyte differentiation need to be further investigated. Although the consequences of lipid accumulation were not investigated here, we hypothesize that the increased intracellular lipid content seen in preadipocytes provides additional substrate for the synthesis of PGE_2_ and other lipid mediators, thus potentiating the inflammatory and vascular effects of this mediator following stimulation with Bmv. The mechanisms underlying Bmv-induced lipid accumulation/adipogenesis in preadipocytes will be the subject of future studies by our group. While there has been a report that bee venom can suppress differentiation of 3T3-L1 preadipocytes, this is to the best of our knowledge the first study showing that a snake venom stimulates lipid accumulation in preadipocytes [78].

Cytokines and chemokines are important inflammatory mediators that drive the development and intensity of inflammatory events [79,80]. Our results showing release of the inflammatory cytokines IL-6 and KC/IL-8 after stimulation of preadipocytes with Bmv are consistent with those showing the release of PGE_2_ and support the idea that this venom triggers an inflammatory response in adipose tissue cells. On the other hand, the adipose tissue specific mediators leptin and adiponectin were not released by Bmv in our experimental condition. Although PGE_2_ has been reported as an inducer of leptin production in adipose tissue explants and adipocytes in culture, this modulation was not observed in Bmv-stimulated preadipocytes. IL-6 is a cytokine with pleiotropic effects that on the one hand promotes inflammation by inducing ICAM-1 expression in endothelial cells and the release of MCP-1 by leukocytes [81–83], and on the other modulates the inflammatory process by stimulating release of the IL-10 cytokine and IL-1 receptor antagonist, leading to an M1 to M2 switch in macrophage phenotypes and thus more resolutive-phase macrophages [84–86]. The chemokine KC is the murine analogue of the human IL-8 cytokine and can activate and recruit neutrophils to the inflammation site [87–89]. Several *Bothrops* spp. venoms are known to induce the release of these two cytokines both *in vitro* and in experimental envenomation in animal models [54,90–92]. Moreover, IL-6 and IL-8 levels were also found to be elevated in the blood of children bitten by *Bothrops* spp. [93]. This evidence strongly suggests that the adipose tissue can be a source of these inflammatory mediators in the event of envenomation by *Bothrops* snakes.

In conclusion, the present work has shown for the first time that *B*. *moojeni* snake venom can stimulate adipose tissue cells. When stimulated with this venom, preadipocytes released PGE_2_, IL-6 and KC/IL-8. Bmv-induced PGE_2_ release was dependent on the COX-1, COX-2 and NF-κB pathways. Furthermore, Bmv upregulated COX-2 protein expression and phosphorylation of NF-κB. It is noteworthy that engagement of the PGE_2_ receptor subtype EP1 by PGE_2_ revealed a positive feedback loop for production of this lipid mediator. Taken together, these results point to the adipose tissue as an additional target for Bmv and suggest that adipose tissue cells contribute to *Bothrops* envenomation by acting as a source of inflammatory mediators.

## Supporting information

S1 Fig Effect of distinct pharmacological compounds on viability of 3T3-L1 cells in culture. 3T3-L1 preadipocytes (2 x 10^5^ cells/well) were incubated with DMSO < 1%, or 1 μM SC-560 (COX-1 inhibitor), or 1 μM NS-398 (COX-2 inhibitor), or 10 μM SC-19220 (EP1 receptor antagonist), or 10 μM AH 6809 (EP2 receptor antagonist), or 1 μM L-798,106 (EP3 receptor antagonist), or 10 μM GW 627368X (EP4 receptor antagonist) for 25 h, or 30 μM TPCK (NF-κB inhibitor) for 48 h, or 50 μg / mL SN50 (NF-κB inhibitor) for 26 h.
(TIF)

S2 Fig Effect of Bmv on proliferation of preadipocytes in culture. 3T3-L1 preadipocytes (2 x 10^5^ cells/well) were incubated with Bmv (0.5 or 1 μg/mL) or DMEM (control) for 24 h. Cell proliferation was assessed by Cell Trace CFSE Cell Proliferation Kit and fluorescence of single cells from a cell population was measured by High-Content Screening (HCS) assay. (A) Representative images of cell proliferation (fluorescence) obtained by HCS (scale bar: 50 μM). Blue: cell nuclei; Green: cell cytoplasm of Bmv-treated cells and DMEM (negative control). (B) Average intensity of Cytoplasmic CFSE / 16 sites analysed.
(TIF)
