# Supplementary figures and images for: Bothrops moojeni snake venom induces an inflammatory response in preadipocytes: Insights into a new aspect of envenomation
Source: PLoS Negl Trop Dis. 2022 Aug 8;16(8):e0010658. doi: 10.1371/journal.pntd.0010658 (PMC9359566; doi:10.1371/journal.pntd.0010658)

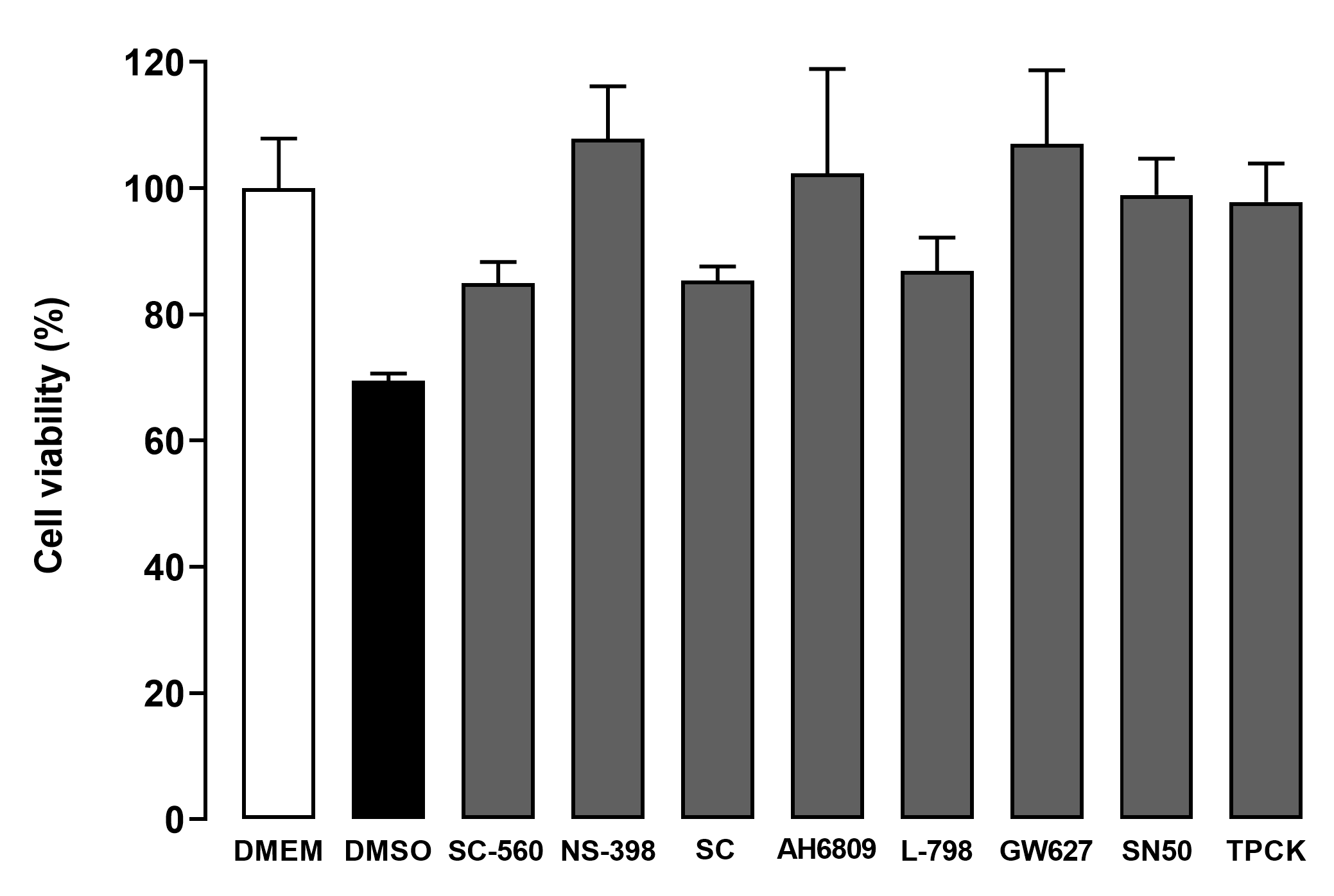

Supplement: S1 Fig — 3T3-L1 preadipocytes (2 x 10^5^ cells/well) were incubated with DMSO < 1%, or 1 μM SC-560 (COX-1 inhibitor), or 1 μM NS-398 (COX-2 inhibitor), or 10 μM SC-19220 (EP1 receptor antagonist), or 10 μM AH 6809 (EP2 receptor antagonist), or 1 μM L-798,106 (EP3 receptor antagonist), or 10 μM GW 627368X (EP4 receptor antagonist) for 25 h, or 30 μM TPCK (NF-κB inhibitor) for 48 h, or 50 μg / mL SN50 (NF-κB inhibitor) for 26 h. (TIF) [file pntd.0010658.s001.tif]

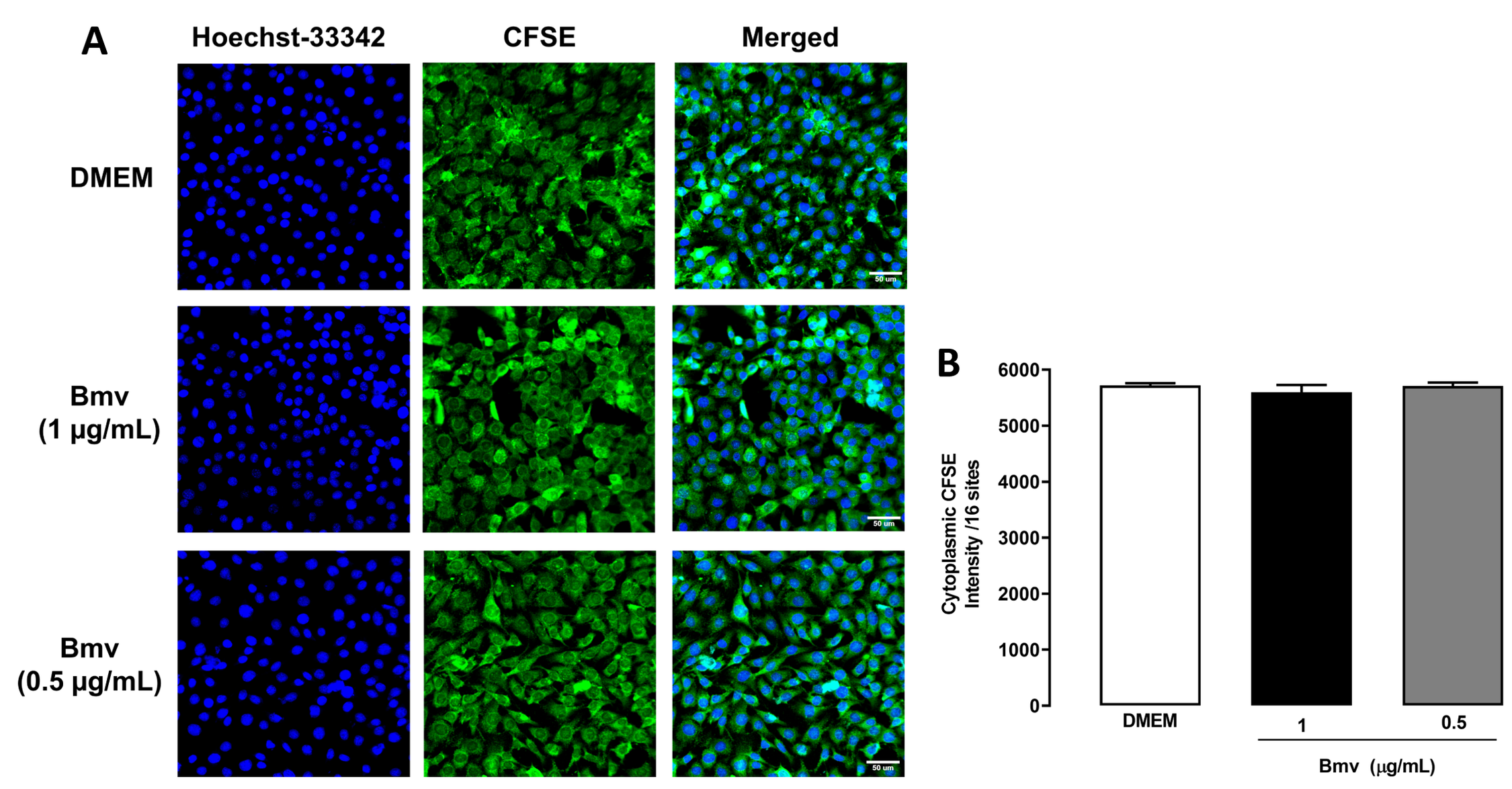

Supplement: S2 Fig — 3T3-L1 preadipocytes (2 x 10^5^ cells/well) were incubated with Bmv (0.5 or 1 μg/mL) or DMEM (control) for 24 h. Cell proliferation was assessed by Cell Trace CFSE Cell Proliferation Kit and fluorescence of single cells from a cell population was measured by High-Content Screening (HCS) assay. (A) Representative images of cell proliferation (fluorescence) obtained by HCS (scale bar: 50 μM). Blue: cell nuclei; Green: cell cytoplasm of Bmv-treated cells and DMEM (negative control). (B) Average intensity of Cytoplasmic CFSE / 16 sites analysed. (TIF) [file pntd.0010658.s002.tif]
